# Carriage of Meningococci by University Students, United Kingdom

**DOI:** 10.3201/eid1709.101762

**Published:** 2011-09

**Authors:** Dlawer A.A. Ala’Aldeen, Neil J. Oldfield, Fadil A. Bidmos, Noha M. Abouseada, Nader W. Ahmed, David P.J. Turner, Keith R. Neal, Christopher D. Bayliss

**Affiliations:** Author affiliations: University of Nottingham, Nottingham, UK (D.A.A. Ala’Aldeen, N.J. Oldfield, N.M. Abouseada, N.W. Ahmed, D.P.J. Turner, K.R. Neal);; University of Leicester, Leicester, UK (F.A. Bidmos, C.D. Bayliss)

**Keywords:** Neisseria meningitidis, epidemiology, meningococcal vaccine, bacteria, United Kingdom

**To the Editor:** *Neisseria meningitidis* causes septicemia and meningitis (*1*). Meningococci usually persist on the nasopharyngeal mucosa of asymptomatic carriers (*2*). Because carriers are the only reservoir of meningococci, carriage in at-risk populations should be monitored. Meningococcal carriage rates have been assessed during 1997–8 for first-year students at the University of Nottingham (*3*) and in autumn during 1999–2001 for >48,000 sixth-form students (pre-university, age range 15–17 years) throughout the United Kingdom (*4*). Serogroup B and nongroupable strains predominated; serogroup Y strains were found in only 1%–2% of participants.

From November 2008 through May 2009, to investigate persistence and spread of meningococcal strains in students living in dormitories, we conducted a longitudinal study in a cohort of 190 first-year students at the University of Nottingham. We found high rates of carriage and prevalence of serogroup Y strains (*5*).

During September 2009 (first week of term) through March 2010, we conducted a large repeated cross-sectional study analyzing pharyngeal swabs from students in all school-year groups at Nottingham University. The objective of this study was to determine the significance of changes in overall meningococcal and serogroup Y-specific carriage rates among students.

In September, first-year students were recruited on the main campus during registration and subsequently in dormitories and the main library. Undergraduates not in the first year were all recruited in the main library. This September sample of 823 first-year students represents 16.5% of the 5,000 undergraduate students registered each academic year on the main campus. Although not intentional, some overlap occurred when students were resampled during subsequent visits to the same dormitories and library, e.g., among the 557 first-year students from whom swab samples were collected in December, 74 (13%) had previously provided swab samples. Our study was approved by the Nottingham University Medical School Ethics Committee, and written informed consent was obtained from all participants.

Pharyngeal swab samples were spread onto GC selective agar (Oxoid, Basingstoke, UK) and incubated at 37°C in air containing 5% CO_2_. After 48 hours, colonies suggestive of *Neisseria* spp. were examined for positive oxidase reaction; single colonies were confirmed as meningococci by amplification of meningococcal genes *crgA* plus *ctrA* and/or *porA* (*6*). PCR-based serogrouping was performed as described (*6**,**7*). Chi-square tests for significance were performed by using STATCALC (Epi Info version 6.04; Centers for Disease Control and Prevention, Atlanta, GA, USA).

Among first-year students, carriage rates increased from 23.2% in late September to 55.7% by mid-December and remained at a similar level in March (Table). Among second- and third-year students, carriage rates were 34.2% and 30.5% in September, respectively, and remained at similar levels throughout the academic year. The increase in carriage among first-year students from September through December was mainly the result of a significant (23%) increase in carriage of serogroup Y strains (Table). In contrast, during the same period, carriage rates of serogroup Y strains did not change significantly among second- and third-year students (Table).

**Table:** Characteristics of meningococci carriage, University of Nottingham students, United Kingdom, 2009–10*

Collection date/year and group	Carriage rate, no. (%) carriers	Serogroup distribution							
		B			Y			Others	
No. isolates (% carried strains)	% All participants (95% CI)	No. isolates (% carried strains)	% All participants (95% CI)	No. isolates (% carried strains)	% All participants (95% CI)
September 2009									
First, n = 823	191 (23.2)†	58 (30.3)	7.0 (5.3–8.8)		24 (12.6)	2.9 (1.8–4.1)‡		109 (57.1)	13.2 (10.9–15.6)
Second, n = 441	151 (34.2)†	34 (22.5)	7.7 (5.2–10.2)		46 (30.5)	10.4 (7.6–13.3)§		71 (47.0)	16.1 (12.7–19.5)
Third, n = 321	98 (30.5)†	35 (35.7)	10.9 ( 7.5–14.3)		20 (20.4)	6.5 (3.6–8.9)¶		43 (43.9)	13.4 (9.7–17.1)
December 2009									
First, n = 557	310 (55.7)#	53 (17.1)	9.5 (7.1–12.0)		142 (45.8)	25.5 (21.9–29.1)‡		115 (37.1)	20.6 (17.3–24.0)
Second, n = 312	123 (39.4)#	33 (26.8)	10.6 (7.2–14.0)		32 (26.0)	10.3 (6.9–13.6)§		58 (47.2)	18.6 (14.3–22.9)
Third, n = 180	52 (28.9)#	12 (23.1)	6.7 (3.0–10.3)		11 (21.2)	6.1 (2.6–9.6)¶		29 (55.8)	16.1 (10.7–21.5)
March 2010									
First, n = 379	224 (59.1)#	44 (19.6)	11.6 (8.4–14.9)		64 (28.6)	16.9 (13.1–20.7)‡		116 (51.8)	30.6 (26.0–35.2)
Second, n = 187	69 (36.9)#	17 (24.6)	9.1 (5.0–13.2)		25 (36.2)	13.4 (8.5–18.3)§		27 (39.1)	14.4 (9.4–19.5)
Third, n = 112	37 (33.0)#	13 (35.1)	11.6 (5.7–17.5)		5 (13.5)	4.5 (0.6–8.3)¶		19 (51.4)	17.0 (10.1–23.9)

*CI, confidence interval. †Carriage rate significantly lower for first-year students than for other year-group students, p <7 × 10^–2^. ‡First-year students, p<8 × 10^–7^. §Second-year students, not significant. ¶Third-year students, not significant. #Carriage rate significantly higher for first-year students than for other year-group students in December 2009 and March 2010, p<10^–8^.

Initial carriage rates were significantly higher for incoming (first-year) students in September 2009 than in 1997 (13.9% [*3*]; χ^2^ = 14, 1 df; p<0.0001); swabbing and culture protocols and sampling sites were identical in both studies, so the increases are real. Because 83% of students at Nottingham University come from all regions of the United Kingdom and 17% from other countries, the increased rates of carriage may reflect a nationwide change (*8*).

Furthermore, testing within the first week of term meant that recovered strains were predominately brought into the university. Serogroup Y carriage rates for incoming students (2.9%) were significantly higher than rates detected by identical genotyping methods during 1999–2001 (1.7%–1.8% [*4*]; χ^2^ = 4.6%–6.4%, 1 df; p<0.05), suggesting that meningococcal carriage by young adults, particularly of serogroup Y strains, has increased across the United Kingdom. The major increase in serogroup Y strains among first-year students during 2009–10 probably resulted from spread of clones within dormitories, as observed in the 2008–9 study (*5*) and may be facilitated by characteristics of the organism, lack of immunity, or a combination of these factors.

The high prevalence of serogroup Y strains in carriers may help explain the recent increased incidence of serogroup Y disease in the United Kingdom: from 20 to 62 laboratory-confirmed cases in England and Wales from 2003 through 2009 (*9*). In the United States during the late 1990s, a similar increase in serogroup Y carriage was linked to a concomitant increase in serogroup Y disease (*10*).

In conclusion, in a representative UK student cohort we detected high rates of carriage and elevated prevalence of serogroup Y strains of meningococci. Any further significant increase in serogroup Y disease should lead to prompt reconsideration of the current vaccine policy in the United Kingdom.
